# Would you believe an intoxicated witness? The impact of witness alcohol intoxication status on credibility judgments and suggestibility

**DOI:** 10.3389/fpsyg.2022.983681

**Published:** 2022-09-30

**Authors:** Georgina Bartlett, Julie Gawrylowicz, Daniel Frings, Ian P. Albery

**Affiliations:** ^1^Centre for Addictive Behaviours Research, Division of Psychology, London South Bank University, London, United Kingdom; ^2^Division of Psychology and Forensic Sciences, Abertay University, Dundee, United Kingdom

**Keywords:** alcohol intoxication, memory conformity, eyewitness memory, intoxicated witness, witness credibility

## Abstract

Memory conformity may occur when a person’s belief in another’s memory report outweighs their belief in their own. Witnesses might be less likely to believe and therefore take on false information from intoxicated co-witnesses, due to the common belief that alcohol impairs memory performance. This paper presents an online study in which participants (*n* = 281) watched a video of a mock crime taking place outside a pub that included a witness either visibly consuming wine or a soft drink. Participants then read a statement from the witness that varied in the number of false details it contained before being asked to recall the crime. We found that the intoxicated witness was regarded as significantly less credible, but participants were not less likely to report misinformation from them. This suggests that intoxication status impacts one’s perception of how credible a source is, but not one’s ability to reject false suggestions from this source. Our findings reinforce the importance of minimizing co-witness discussion prior to interview, and not to assume that people automatically (correctly or not) discount information provided by intoxicated co-witnesses.

## Introduction

Studies show that police officers report routinely encountering witnesses who have consumed alcohol (Crossland et al., 2018). Intoxicated witnesses are as likely to make a suspect identification as their sober counterparts and to give a statement to police (Palmer et al., 2013). On this basis, research has focused on elucidating the effects of alcohol intoxication on eyewitness memory accuracy. In laboratory studies using moderate doses of alcohol, studies suggest that intoxicated witnesses are less complete in their recall (Schreiber Compo et al., 2011), but also no less accurate (Flowe et al., 2019) and no more prone to reporting false information (Compo et al., 2012; Flowe et al., 2019; Bartlett et al., 2021, for a meta-analysis, see Jores et al., 2019). Also, intoxicated witnesses show least impairment in their recall accuracy and are less prone to suggestibility when recalling immediately compared to after a delay (Evans et al., 2017; Schreiber Compo et al., 2017). At moderate doses there also appears to be a benefit of using free recall formats over cued recall questions to reduce the reporting of incorrect details (Compo et al., 2012; Schreiber Compo et al., 2017). At higher doses, the quality of memory reports starts to decline (Altman, et al., 2018) and witnesses may become more prone to suggestibility (Van Oorsouw et al., 2015, 2019), although these findings are equivocal (Crossland et al., 2016).

It appears, therefore, that while there is evidence that intoxication impairs the quantity of memory recall, the quality of memory reports is maintained, at least at moderate doses. This conclusion is inconsistent with the perception people often have of witnesses who have consumed alcohol. For instance, in work examining juror perceptions of witnesses who are intoxicated by alcohol or drugs, juror-eligible participants reported believing that alcohol has a large negative effect on memory and that intoxicated witnesses are less credible than sober ones (Monds et al., 2021). In addition, Crossland et al. (2021) showed that knowledge of a witness’ prior intoxication and less completeness of their account led to lower-rated credibility. As juries consist of lay people, their perceptions and judgments can be considered indicative of a common-sense belief. This perception extends to professionals within the criminal justice system. For example, Sleath and Bull (2017) showed that police officers judged rape victims who were intoxicated as less credible and as having engaged in risk-taking by consuming alcohol. In addition, an intoxicated victim of rape was more likely to be blamed for an incident occurring than a sober one (Rape Crisis Network Ireland, 2020). In essence as perceived victim intoxication increases, both their perceived credibility and blame for the perpetrator decreases, while victim-blame increases (Schuller and Stewert, 2000). There appears to be a disparity between the *credibility* of an intoxicated witness, and their *reliability* as a source of information (Porter and Brinke, 2009). While a witness can be deemed credible despite their testimony being unreliable, in the case of intoxicated witnesses it appears the reverse may be true-despite often being reliable, they are perceived as not being credible among judges, jurors, and justice system professionals.

The finding that an intoxicated witness is not perceived as a credible source may influence the likelihood of reporting misinformation from them. Memory conformity studies have shown that when the source of information is less credible than the person receiving it, the tendency to report misinformation is reduced (Gabbert et al., 2007; French et al., 2011). This suggests that in scenarios where there are multiple witnesses to a crime, a person may be less likely to report misinformation gained from an intoxicated source. In one study, while intoxicated participants were no more likely to report contagion items proposed by a confederate than sober participants, they were less likely to take on information from a confederate they perceived to be under the influence of alcohol (Thorley and Christiansen, 2018). The perceived “intoxicated” confederate was also viewed as less accurate and trustworthy compared to the sober one. In other work, when the misinformation proposed by a confederate was discrepant with the participants’ own judgment, participants were less likely to take on the information proposed by an intoxicated confederate relative to a sober confederate (Zajac et al., 2016). It seems that perceived intoxication appears to make a co-witness less credible which, in turn, means that their discussion partner might be less likely to take on erroneous information from them.

The present study addressed whether intoxication and the number of misinformation items reported by a source influences the likelihood of a mock-witness to take on information from that person and subsequently incorporate it into their personal account of the event. Previous studies have identified several factors that influence the tendency to take on information from a co-witness including relative visual acuity and confidence (Gabbert et al., 2007; French et al., 2011; Goodwin et al., 2013). Work in this area has shown that individuals are just as likely to report misinformation from a source that is mostly accurate as they are from a source that was completely inaccurate (Numbers et al., 2014). Other evidence suggests that the tendency to report misinformation is reduced when participants knew that a discussion partner was of low credibility (Andrews and Rapp, 2014). Thus, perceived co-witness credibility appears to be more influential than actual credibility when it comes to being susceptible to memory conformity.

The present study manipulated the intoxication of the co-witness to test for differences in perceived credibility. If intoxicated witnesses were perceived as less credible then participants should be less likely to report misinformation from the intoxicated co-witness (see Andrews and Rapp, 2014). Furthermore, the number of erroneous details (low versus high) in the co-witness report was manipulated to examine the impact of actual credibility on participants’ accounts and their likelihood to take on misinformation. We were also interested in how perceived intoxication status and the number of erroneous details provided would interact and impact one’s susceptibility to report misinformation. We also collected confidence data from participants for each of their responses as previous work has shown participants to be less confident when their answers included misinformation (Gabbert et al., 2003). The present study examined whether participants were less confident in their response to specific questions pertaining to misinformation (rather than details gained from the video).

Based on the accumulated evidence discussed earlier, we examined predicted relationships through the computation of a moderated mediation model. This model identified whether credibility ratings of the co-witness given by participants mediated the relationship between co-witness intoxication and reporting of misinformation (see Thorley and Christiansen, 2018). Using this approach, we examined whether participants were always less likely to report misinformation from an intoxicated co-witness or whether this tendency was related to the credibility ratings they attributed to the co-witness. In addition, we included the number of errors in the co-witness’ statement as a moderator such that reporting of misinformation was only possible when participants read a statement containing misinformation.

Based on the evidence outlined above, we proposed the following hypotheses:

- Participants would report significantly less misinformation from an intoxicated co-witness than from a sober witness (Zajac et al., 2016; Thorley and Christiansen, 2018)
- Participants would rate an intoxicated witness as less credible than a sober witness (see Wall and Schuller, 2006; Evans et al., 2017)
- Participants would report less misinformation from the intoxicated co-witness who reported a high number of errors than the intoxicated co-witness who reported a low number of errors (Andrews and Rapp, 2014)
- Participants would be significantly less confident when their answer included misinformation than when it does not (Gabbert et al., 2003).

## Methods

### Design

The study used a 3 × 2 between-subjects design in which participants were randomly assigned to one of six experimental conditions. The between-subject factors were intoxication status (intoxicated vs. sober) and statement errors (no errors vs. low errors vs. high errors). The dependent variables were reported misinformation and confidence scores on the cued recall questionnaire. The study received ethical approval from the Ethics Panel at London South Bank University.

### Participants

Two hundred eighty-one participants took part in the study (*M*age = 25.84 years, SD = 9.62, range = 18–62). They were recruited *via* the university research participation scheme system (course credit for participation) and *via* social media (i.e., Twitter, Facebook, and Reddit). Two hundred and nine participants were female, 46 were male, and 3 indicated that they preferred not to say, while 23 did not provide an answer. Achieved power analysis on the lowest *R* squared achieved (0.14) with a Cohen’s *f* squared of 0.16 (a medium effect) demonstrated that a sample of 281 participants, with 4 predictors had a power of 0.99.

## Materials

### Videos

Participants were randomly assigned to one of two videos. Both videos depicted an incident outside a pub, and each showed the same sequence of events: a male and female talking, after which a second male attempts to get past them, pushes the first male, and walks away. The key difference between the videos was the intoxication status of the female witness. In the “intoxicated” version the witness is seen ordering a glass of wine from the bar prior to meeting her friend outside the pub and is later shown stumbling and slurring her words. In the “sober” video version, the female is seen ordering a glass of orange juice and mentioning to her friend that she is driving and can offer him a lift home.

### Vignettes

The study utilized six written vignettes, purportedly written by the female witness, as a statement to the police about the incident. The written scenarios differed in two ways. First, depending on the video participants watched, the witness reported either having drunk three glasses of wine or that she had decided to drive and so was drinking orange juice. Secondly, they differed in the number of errors reported: no errors, two errors (low error condition), or four errors (high error condition). Table 1 presents four details present in the “no errors” condition with their corresponding details in the “low” and “high” errors condition.

**Table 1:** Key details included in the witness statements in the no errors, low errors, and high errors conditions.

Witness scenario		
No errors	Low errors	High errors
Attacker had brown hair	Attacker had brown hair	Attacker had shaven hair*
Attacker wore green top	Attacker wore black hoodie*	Attacker wore black hoodie*
Boyfriend apologies for		
bumping into attacker	Boyfriend apologies for bumping into attacker	Attacker swore at witness*
Victim was pushed	Victim was pushed and kicked*	Victim was pushed and kicked*

Errors are indicated by *.

### Memory tests

A cued recall questionnaire was used to assess participants’ memory of the video content. Questions related to the appearance of the witness and the assailant as well as to details of the crime. Five questions were classed as “neutral” (i.e., those that ask about details for which participants did not receive misinformation, including “where were the victim and the witness?”) whilst four questions were categorised as “critical” (i.e., those that asked about details for which participants may have received misinformation, including “what was the attacker wearing?”). Participant responses were coded as “correct” (if the response correctly described the events in the video,) “error” (if video events were incorrectly described), “misinformation” (if the response used post event information (PEI)) from the vignette, or “I do not know” (if the participant reported that they could not remember the detail). For each question participants were also asked to indicate confidence in their responses on type scales ranging from “not at all confident” (scored as 1) to “extremely confident” (scored as 4).

### Intoxication and credibility assessment

Participants were asked to rate “(H)ow drunk do you think the witness was?” with the options of “completely sober” “mildly intoxicated” and “very intoxicated” as well as “(H)ow credible do you think the witness was?” with response options “not at all credible” “reasonably credible” and “very credible”.

### Procedure

The present study was completed individually and online. After consenting, participants were randomly presented with the video including either the intoxicated witness or the sober one. Participants were instructed that they would be viewing a video of an incident that occurred outside a pub, before reading a statement that the witness gave to the police. They then read one of the six “witness statements” depending on their randomized condition. The written scenario always matched the video such that those who viewed the intoxicated video version were presented with the intoxicated witness statement and those who watched the sober video version were presented with the sober witness statement. After reading the witness statement participants were asked to complete a 10-min “spot the difference” task as a filler activity, and then completed the cued recall test about the video. Participants were asked to answer the questions about the events in the video as accurately as possible, they then provided perceptions of witness intoxication, and witness credibility.

## Results

### Intoxication and credibility assessment

Participants who saw the video containing the presumably intoxicated witness rated the witness as significantly more drunk (*M* = 2.20, *SD* = 0.44) than participants who viewed the video containing the presumably sober witness (*M* = 1.24, *SD* = 0.46, 95%) CIs [−1.06, −0.85]; *t* (273) = 17.73, *p* < 0.001, *d* = 2.14, suggesting that the alcohol intoxication status manipulation was successful.

To investigate whether participants’ credibility ratings differed based upon the accuracy of the witness’ statement and the presence of alcohol, a two-way ANOVA was used. A significant effect of statement errors was found *F* (2,265 = 13.92, *p* < 0.001, ηp^2^ = 0.095). Participants in the no errors condition perceived the witness as significantly more credible (*M* = 2.07, *SE* = 0.06) than in the high errors condition (*M* = 1.64, *SE* = 0.07, *p* < 0.001, 95% CIs [0.26–0.60]). Participants who read the low errors statement also perceived the witness as significantly more credible (*M* = 2.02, *SE* = 0.06) than those who read the high errors statement (*p* < 0.001, 95%) CIs [0.21–0.56]. There was no significant difference in credibility between those who read the no errors and low errors statements (*p* = 1.00, 95%) CIs [−0.18, 0.23]. A significant effect of intoxication was also found *F* (1,265 = 27.20, *p* < 0.001, ηp^2^ = 0.093). Participants who viewed the witness consuming alcohol perceived them as significantly less credible (*M* = 1.72 *SE* = 0.05) than participants who viewed the witness drinking orange juice (*M* = 2.09, *SE* = 0.05), 95% CIs [0.230–0.508]. There was no significant interaction between statement errors and intoxication *F* (2,265) = 0.92, *p* = 0.399, ηp^2^ = 0.007. In sum, participants’ judgments of the witness’ credibility changed according to the number of errors in her statement, with the statement containing most errors being perceived as less credible. Additionally, when the witness was presumably intoxicated, she was also perceived as less credible. However, there was no interaction between statement errors and intoxication status on the perception of witness credibility.

### Misinformation analysis

**Figure 1:**
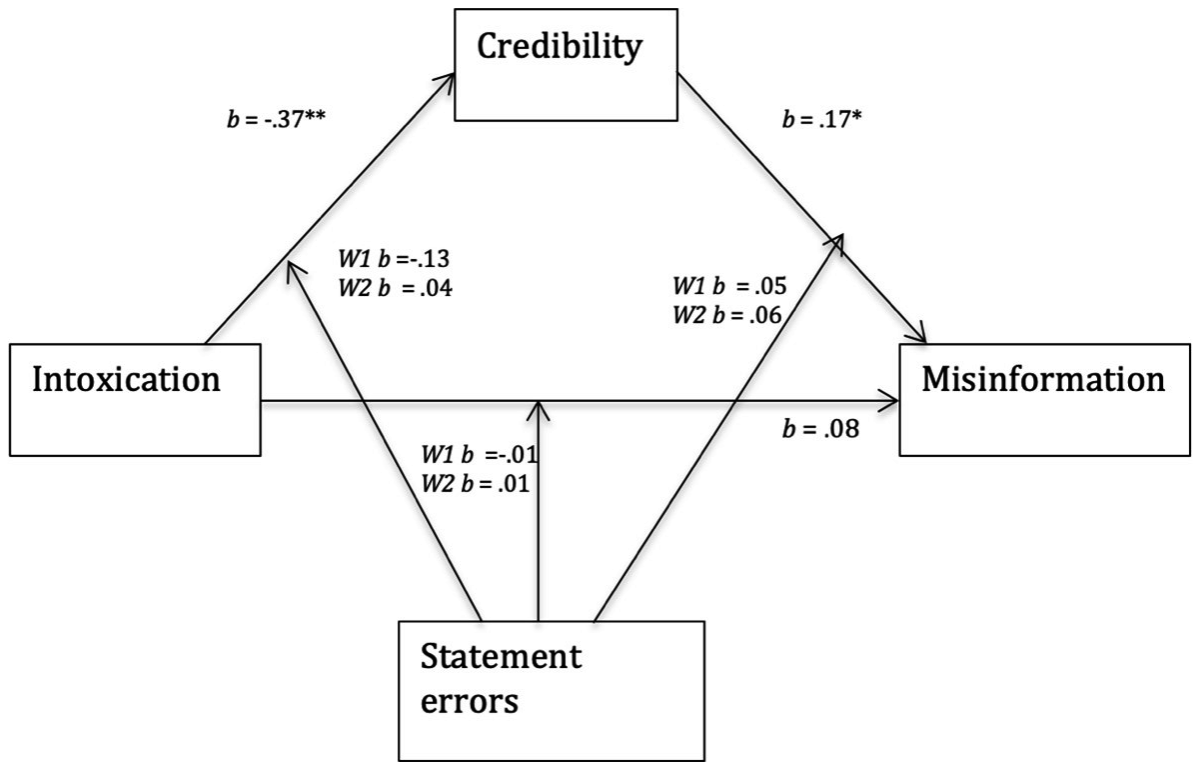
Moderated mediation model examining the mediating effect of perceived credibility on the relationship between witness intoxication and the tendency to report misinformation. Additionally, whether this mediating effect is moderated by the number of statement errors, at low vs. no (W1) and high (W2) levels. * Denotes significance at *p* = 0.01, ** denotes significance at *p* = 0.001.

To examine whether participants would be less likely to take on misinformation from an intoxicated witness a moderated mediation model was computed (see Figure 1). This model was computed using model 59 of the regression-based Process SPSS plug-in (Hayes, 2018, version 3.4) incorporating the bootstrapping of 1,000 samples. The model examined the mediating effect of perceived credibility on the relationship between intoxication and the tendency to report misinformation and whether this mediation effect is moderated by statement errors. The mediation model chosen examined the number of statement errors as a potential moderator between intoxication and perceived credibility, intoxication and misinformation, and perceived credibility and misinformation.^1^

“Statement Errors” was a multi-categorical variable and as such was re-coded into two *dummy* variables: *W*1 concerns the effect of low errors compared to no errors and *W*2 compares the effect of high errors relative to no errors. An achieved power analysis based upon the observed zero order correlations between predictor, dependent variable and mediator indicated that a model with one predictor and one mediator and a sample of 281 participants had a power of 0.18 to detect indirect mediation effects. Further power analysis showed that the sample would reach a power of 0.8, if the smallest correlation of −0.007 (between dependent variable and predictor) increased to −0.60. In sum, the sample was arguably sufficient to detect mediation effects under conditions they would be likely to manifest. The model predicting perceived credibility was significant *F* (5, 2,695) = 11.18, *p* < 0.001, *R*^2^ = 0.17. Intoxication and statement errors predicted 17% of the variance in credibility ratings and were both significant independent predictors of credibility. The interactions between intoxication and statement errors were not significant (W1: *b* = −0.13 *t*(265) = 1.30, *p* = 0.19; W2: *b* = 0.04, *t*(265) = 0.40, *p* = 0.69) suggesting that statement errors did not moderate the relationship between intoxication and perceived credibility.

The model predicting the tendency to report misinformation was significant *F* (8, 262) = 5.28, *p* < 0.001, *R*^2^ *=* 0.14 (Figure 1 and Table 2). Perceived credibility and being in the high error statement condition were significant independent predictors in the model. However, intoxication was not a significant predictor of misinformation. The interactions between credibility and statement errors were not significant nor were the interactions between intoxication and statement errors. In sum, statement errors independently predicted the incorporation of misinformation but did not moderate the relationship between credibility and misinformation nor intoxication and misinformation. There were no direct effects of intoxication on the tendency to report misinformation (*p*s > 0.05) nor indirect effects of intoxication on the tendency to report misinformation (*p =* 0.309). As such, the intoxication of the co-witness did not predict participants’ susceptibility to misinformation. Additionally, perceived credibility did not mediate the relationship between co-witness intoxication and susceptibility to misinformation.

**Table 2:** Regression coefficients for predicting misinformation from perceived credibility, witness intoxication and statement errors.

	B	SE B	*t*	sig
Intoxication	0.08	0.08	1.01	0.31
Perceived credibility	0.17	0.07	2.60	0.01
Low errors (W1)	−0.10	0.06	−1.74	0.08
High errors (W2)	0.36	0.06	5.94	<0.001
Intoxication × W1	−0.009	0.12	−0.08	0.94
Intoxication × W2	0.01	0.12	0.11	0.92
Perceived credibility × W1	0.05	0.10	0.53	0.60
Perceived credibility × W2	0.06	0.09	0.69	0.49

### Accuracy

To examine whether there was an effect of statement errors or witness intoxication status on participants’ accuracy rate a two-way ANOVA was used. Accuracy rate was computed by dividing the number of accurately reported details in the cued recall by the total number of details (see Table 3 for average reporting of details between conditions). There was no significant main effect of intoxication (*F* (1, 275) = 0.55, *p* = 0.458, ηp^2^ = 0.002) or statement errors on accuracy rate (*F* (2,275) = 2.25, *p* = 0.107, ηp^2^ = 0.016), nor a significant interaction (*F* (2,275) = 0.87, *p* = 0.421, ηp^2^ = 0.006). Therefore, other than the propensity to report misinformation, there were no differences between conditions in the accuracy of information reported.

**Table 3:** Means (standard deviations in parentheses) of response type within each condition.

Response type						
		Correct	Error	Misinformation	IDK	Accuracy rate
Sober witness	No errors	6.63 (2.13)	1.50 (1.20)	0.13 (0.35)	0.75 (1.04)	0.81 (0.15)
	Low errors	7.00 (1.73)	0.93 (1.10)	0.33 (0.48)	0.73 (0.88)	0.78 (0.14)
	High errors	5.50 (2.37)	1.80 (1.87)	0.80 (1.03)	0.90 (1.10)	0.73 (0.23)
Intoxicated witness	No errors	6.33 (2.00)	1.11 (1.05)	0.00	1.56 (2.30)	0.78 (0.20)
	Low errors	5.90 (2.38)	0.90 (1.20)	0.10 (0.32)	2.10 (2.64)	0.75 (0.19)
	High errors	6.90 (1.70)	1.10 (1.04)	0.55 (1.04)	0.37 (0.50)	0.76 (0.17)

### Confidence

To investigate whether participants would be less confident in their responses when they incorporated misinformation a 3 × 2 × 3 mixed ANOVA was used with participant response (correct, misinformation, error), intoxication status (sober vs. intoxicated), and statement errors (no errors, low errors, high errors) as the independent variables. The results indicated a significant effect of response type on confidence ratings *F*(2, 78) = 14.87, *p* < 0.001, ηp^2^ = 0.27. Participants were significantly less confident when they responded with an error (EMM = 1.85, SE = 0.10) than correct information (EMM 2.64, SE = 0.11). Participants were also significantly less confident when they responded with an error than with misinformation gained from the co-witness statement (EMM = 2.51, SE = 0.15). There was no significant main effect of intoxication (*F*((1.39)) = 0.21, *p* = 0.65, ηp^2^ = 0.005) or statement errors (*F*((2,30)) = 0.40, *p* = 0.67, ηp^2^ = 0.02). There was a significant interaction between response type and statement errors *F*(4, 78) = 3.36, *p* = 0.014, ηp^2^ = 0.15. In all statement error conditions confidence was higher for correct and misinformation responses than incorrect responses. This difference was greatest in the “low errors” statement errors condition. There was also a significant interaction between intoxication and statement errors on participant confidence *F*(2, 39) = 7.14, *p* = 0.002, ηp^2^ = 0.27. Simple effects analyses indicated that for participants who viewed a sober witness, confidence was significantly higher in the low errors condition than in the high errors condition (*p* = 0.039) whereas, for participants who viewed an intoxicated witness, there was no significant difference between error conditions (*ps* > 0.05).

As such, contrary to the hypothesis, participants were not significantly less confident when they responded with misinformation than when they responded correctly. Furthermore, participants were significantly more confident when they responded with misinformation than when they responded incorrectly.

To further investigate the effects of statement errors, intoxication status and question type on confidence a 2 × 2 × 3 mixed ANOVA was used with question type (neutral vs. critical), intoxication status (sober vs. intoxicated) and statement errors (no errors, low errors, high errors) as the independent variables. The results indicated a significant effect of question type on confidence ratings (*F*(1,278) = 211.6, *p* < 0.001, ηp^2^ = 0.436). Participants were significantly more confident in their response to neutral questions than in response to critical questions. There was no significant main effects of statement errors (*F*(1,274) = 0.58, *p* = 0.562, ηp^2^ = 0.004) or intoxication status (*F*(2, 274) = 1.5, *p* = 0.223, ηp^2^ = 0.005) nor significant interactions between statement errors and question type (*F*(1,278) = 0.001, *p* = 0.144, ηp^2^ = 0.014) or question type and intoxication status (*F*(1, 274) = 2.41, *p* = 0.122, ηp^2^ = 0.009). However, the three-way interaction between question type, intoxication status and statement errors was significant (*F*(2, 274) = 4.83, *p* = 0.009, ηp^2^ = 0.034).

To break down this three-way interaction, interactive and simple effects of intoxication status and question type were calculated at each level of the statement errors condition.

For participants in the “no errors” statement condition, there was a significant main effect of question type on confidence, *F*(1, 101) = 78.36, *p* < 0.001, ηp^2^ = 0.44. Participants were significantly more confident in response to neutral questions (*M* = 3.04, SD = 0.67) than critical questions (*M* = 2.65, SD = 0.64). There was no significant main effect of witness intoxication on participant confidence, *F*(1,101) = 1.78, *p* = 0.185. However, there was a significant interaction between witness intoxication and question type, *F*(1, 101) = 7.43, *p* = 0.008, ηp^2^ = 0.07. When the witness was sober, the difference in participant confidence in response to neutral vs. critical questions was greater than when the witness was intoxicated.

For participants in the “low errors” statement condition there was a significant main effect of question type, *F*(1, 95) = 94.97, *p* < 0.001, ηp^2^ = 0.50. Participants again reported higher confidence in response to neutral (*M* = 3.05, SD = 0.67) than critical questions (*M* = 2.55, SD = 0.59). There was no significant main effect of witness intoxication or significant interaction (*p*s > 0.05).

For participants in the “high errors” statement condition, there was a significant main effect of question type on participant confidence, *F*(1, 78) = 45.55, *p* < 0.001, ηp^2^ = 0.37. Once again, participants were significantly more confident in response to neutral (*M* = 2.94, SD = 0.74) than critical questions (*M* = 2.56, SD = 0.67). There was no significant main effect of witness intoxication, or significant interaction (*ps* > 0.05).

Thus, for all participants, confidence was higher in response to neutral questions than critical ones. For participants who did not receive any misinformation, confidence was higher in response to neutral questions compared to critical ones, but only when the witness was sober.

## Discussion

This study examined whether participants would be less likely to report misinformation from an intoxicated co-witness than a sober one due to a belief that they were less credible. The results showed that participants did in fact view the intoxicated co-witness as less credible than the sober co-witness. However, there was no effect of witness intoxication status on the incorporation of misinformation. Perceived credibility significantly related to the amount of misinformation participants reported but it did not mediate the relationship between intoxication of the witness and misinformation. Participants included significantly more misinformation in the “high statement errors” condition regardless of whether the witness was reportedly sober or intoxicated. Additionally, participants’ confidence in their own responses was significantly higher for neutral questions than critical questions. For participants who did not receive any misinformation, this difference was larger when the witness was sober. This finding is surprising considering that these participants were not exposed to any misinformation. The findings may suggest that all participants found these questions more difficult regardless of encountering misinformation. The finding that confidence was generally lower when participants viewed the intoxicated co-witness in the no errors condition may additionally reflect a lack of trust in the information reported by the intoxicated co-witness despite their account being correct. Future research should investigate the effect of encountering only correct information from both a sober and intoxicated co-witness on witness confidence in their own testimony.

That the intoxicated witness was regarded as less credible than the sober one is consistent with previous research (e.g., Schuller and Stewart, 2000; Kassin et al., 2001; Monds et al., 2021). However, this did not lead to a reduced incidence of reporting misinformation from the source. The literature has demonstrated that intoxicated witnesses and victims are seen as less credible (Schuller and Stewert, 2000) and that the source’s perceived credibility can impact the tendency to report misinformation (Gabbert et al., 2007; French et al., 2011). Hence one would expect that participants are less likely to report such details from the intoxicated source. The context in which one encounters the intoxicated witness may be important (see Mason et al., 2004). Previous studies have examined instances of rape and sexual assault under conditions of intoxication (Evans and Compo, 2010), which are prone to influence by a person’s adherence to, for example, rape myths. Furthermore, juror decision-making studies require participants to appraise the testimony of a witness to decide on a decision of guilt (Lynch et al., 2013). Given these task requirements, the testimony of an intoxicated witness may come under greater scrutiny than in the present study, where no associated consequences for the co-witness, victim, or perpetrator were apparent.

Furthermore, research by Pantazi et al. (2020) investigated the presence of a “truth bias” in mock jurors and judges. Participants are biased to assume the information they receive is true, despite it being identified as false. The mechanism through which this is said to occur is called meta-cognitive myopia. That is, individuals are sensitive to the primary information they are obtaining but may not demonstrate the same sensitivity to additional meta-information that may contain important contextual cues relevant to the accuracy of the material. In addition, Lassiter et al. (2001) report how increasing accountability by informing participants that they will have to justify their decision resulted in more careful processing of information. As such, the tendency to report misinformation despite perceiving the witness as less credible may have arisen due to a combination of truth bias and lack of scrutiny given to the witness statement in a context in which individual accountability is low.

The finding that participants were not less likely to report misinformation when it was encountered from an intoxicated source is inconsistent with previous research (e.g., Zajac et al., 2016; Thorley and Christiansen, 2018). Zajac et al. (2016) found that perceived intoxication reduced memory conformity at an individual item level when the confederate’s response was discrepant with the participants’ initial response. However, consistent with the present study, overall susceptibility to misinformation did not differ based upon co-witness intoxication. In this study, we did not ask participants to provide an initial response prior to the post-event-information exposure by the co-witness. As such, there was no direct explicit discrepancy between the co-witness and the participant that could have further influenced their likelihood to report misinformation. Furthermore, a distractor task preceded the questioning phase giving participants even less opportunity to detect any discrepancies between their own memory and that of the witness. Also, while Thorley and Christiansen (2018) showed a significant effect of perceived intoxication on reporting of misinformation, their use of a social contagion task (i.e., participants recalled household scenes alongside a confederate) is a very different methodology to that incorporated in the present study in which participants were exposed to a written statement containing incorrect details. Importantly, such a recall scenario includes less contextual information, and participants may have been better able to detect erroneous confederate suggestions.

Whether individuals are taking on misinformation from another person might also depend on the participants’ subjective interpretation of the term “credibility.” Williamson et al. (2013) manipulated the expertise of their co-witness (assuming policeman = high expertise vs. electrician = low expertise) and tested whether this would impact credibility ratings and memory conformity. They found that Participants were more likely to take on misinformation from the policeman compared to the electrician. The policeman was also rated as more credible than the electrician. However, perceived credibility did not predict memory conformity but only perceived memory accuracy and memory confidence. So, could it be that participants perceived the intoxicated co-witness as less credible but not necessarily less accurate in our study? To answer this question future studies should provide a more thorough definition of the term credibility to their participants or break the term up into different components, such as memory accuracy and memory confidence, as Williamson et al. (2013) did.

Alternatively, participants may not have believed that the co-witness was sufficiently intoxicated to prevent them from reporting misinformation from the co-witness. Participants’ tendency to report misinformation reported by the intoxicated co-witness may thus be influenced by alcohol expectancies, drawn from one’s own direct and indirect experience of alcohol consumption (Merrill et al., 2016). They may have decided that after consuming three glasses of wine, a witness’ memory would not be greatly impaired. However, that the intoxicated witness was regarded as significantly less credible than the sober counterpart suggests that their intoxication was considered as detrimental to their overall credibility. Future work should compare the likelihood of reporting misinformation from a sober witness, a mildly intoxicated witness, and a heavily intoxicated witness. This would allow to examine whether the likelihood to incorporate misinformation is related to intoxication severity.

Participants may also have reported misinformation because they incorrectly attributed the source of the misinformation to the video rather than to the written scenario (a source misattribution error). The tendency to make such a misattribution is increased when the target and false stimuli are highly similar (Johnson et al., 1993). In the present study, the contextual details used in the witness’ statement, and the similarity between the real version of events and the errors, may have led to a decreased ability to detect the discrepancies between the two (Lapaglia and Chan, 2019). As the present study did not include a source monitoring task, this explanation is tentative given the findings, and it is likely that a combination of normative influence, informational influence, and source monitoring misattributions led to the tendency to report misinformation. Future work should include source monitoring questions to distinguish the effects of these individual mechanisms.

## Limitations and future directions

While the present study established a relationship between alcohol intoxication and credibility, credibility was assessed using a single-item scale. This was chosen to reduce participant attrition which commonly occurs in online studies (Reips, 2000; Zhou and Fishbach, 2016). The single items were sufficient to establish that the alcohol manipulation had resulted in a difference in perceived intoxication and credibility between the two witnesses. It also established that there was a relationship between intoxication status and perceived credibility outside of a juror decision-making setting. In the future, it would be useful to include a questionnaire regarding specific aspects of credibility (like Williamson et al., 2013 did) to gain a more nuanced understanding of the perception of intoxicated witnesses as less credible. In addition, future work should include a source monitoring questionnaire which assesses whether participants gained the information for their responses from the co-witness or the video. This would allow for conclusions to be drawn regarding the mechanisms underlying the incorporation of misinformation, for example, whether misinformation was incorporated due to a source monitoring error or due to informational influences. The study took place online and therefore participants completed it without supervision from the researcher. While such methodology is beneficial in that it allows one to reach a larger sample, it also has its own limitations. Namely, some participants may not have paid close attention to the video which may introduce random errors into the data. Future research may wish to include attention check questions to ensure that all participants have paid sufficiently close attention to the content.

## Conclusion

The present study demonstrated that the perceived accuracy of an intoxicated witness as well as their perceived intoxication influences the tendency to perceive them as credible or not. We also showed that sober participants were just as likely to report false information from a presumably intoxicated witness as a sober one. These findings have implications for the criminal justice system as it suggests that witnesses who are exposed to misinformation prior to giving their statement will be as likely to report such information if it was gained from an intoxicated or a sober witness. Given that the highest proportion of violent crime in England and Wales in the year 2015/16 occurred on Fridays and Saturdays between 9 p.m. and 3 a.m. (Institute of Alcohol Studies, 2017), encountering misinformation from an intoxicated witness is a real risk. The present study adds to the body of work suggesting that, regardless of the intoxication status of witnesses, co-witness discussion should be discouraged where possible to avoid the risk of witnesses reporting information in their own account that they did not actually see or hear.

## Data availability statement

The raw data supporting the conclusions of this article will be made available by the authors, without undue reservation.

## Ethics statement

The studies involving human participants were reviewed and approved by University ethics Panel at London South Bank University. The patients/participants provided their written informed consent to participate in this study.

## Author contributions

The design of the study was developed by all authors, and data was collected by GB. GB and DF conducted the data analysis. GB, JG, and IA wrote the paper. DF and JG provided additional revisions to the style and structure of the paper. All authors contributed to the article and approved the submitted version.

## Conflict of interest

The authors declare that the research was conducted in the absence of any commercial or financial relationships that could be construed as a potential conflict of interest.

## Publisher’s note

All claims expressed in this article are solely those of the authors and do not necessarily represent those of their affiliated organizations, or those of the publisher, the editors and the reviewers. Any product that may be evaluated in this article, or claim that may be made by its manufacturer, is not guaranteed or endorsed by the publisher.

## Footnotes

^1^Two other models were also tested with statement errors as a moderator only between perceived credibility and misinformation, and only between intoxication and perceived credibility. Both models showed a significant effect of perceived credibility and statement errors on misinformation with no interactions.
